# Identification of Novel Therapeutic Targets for Pulmonary Arterial Hypertension

**DOI:** 10.3390/ijms19124081

**Published:** 2018-12-17

**Authors:** Kimio Satoh, Nobuhiro Kikuchi, Taijyu Satoh, Ryo Kurosawa, Shinichiro Sunamura, Mohammad Abdul Hai Siddique, Junichi Omura, Nobuhiro Yaoita, Hiroaki Shimokawa

**Affiliations:** Department of Cardiovascular Medicine, Tohoku University Graduate School of Medicine, Sendai 980-0872, Japan; n-kikuchi@cardio.med.tohoku.ac.jp (N.K.); s-taiju@cardio.med.tohoku.ac.jp (T.S.); r_kurosawa@cardio.med.tohoku.ac.jp (R.K.); ssunamura@cardio.med.tohoku.ac.jp (S.S.); ah.siddique@cardio.med.tohoku.ac.jp (M.A.H.S.); junp0103@cardio.med.tohoku.ac.jp (J.O.); tohokuyaoita@cardio.med.tohoku.ac.jp (N.Y.); shimo@cardio.med.tohoku.ac.jp (H.S.)

**Keywords:** pathogenesis, pulmonary hypertension, biomarker, drug discovery

## Abstract

Pulmonary arterial hypertension (PAH) and chronic thromboembolic pulmonary hypertension (CTEPH) are fatal diseases; however, their pathogenesis still remains to be elucidated. We have recently screened novel pathogenic molecules and have performed drug discovery targeting those molecules. Pulmonary artery smooth muscle cells (PASMCs) in patients with PAH (PAH-PASMCs) have high proliferative properties like cancer cells, which leads to thickening and narrowing of distal pulmonary arteries. Thus, we conducted a comprehensive analysis of PAH-PASMCs and lung tissues to search for novel pathogenic proteins. We validated the pathogenic role of the selected proteins by using tissue-specific knockout mice. To confirm its clinical significance, we used patient-derived blood samples to evaluate the potential as a biomarker for diagnosis and prognosis. Finally, we conducted a high throughput screening and found inhibitors for the pathogenic proteins.

## 1. Introduction

Pulmonary arterial hypertension (PAH) is characterized by histological changes in the distal pulmonary arteries, such as intimal/medial thickening, and perivascular inflammation and fibrotic change, resulting in right ventricular failure and premature death [1]. This often occurs in young women, and initial symptoms are often overlooked. In many cases, patients experience severe right heart failure when visiting a specialized facility. Early diagnosis is difficult, even by cardiovascular specialists, therefore it is already in the terminal stage when introduced to a specialized hospital. In addition to genetic backgrounds, such as mutations in bone morphogenetic protein receptor 2 (*BMPR2*) [2], many environmental factors (e.g., hypoxia [3], infection [4], smoking [5], air pollution [6], daily diet [7], and medications [8]), as well as volume overload, due to heart disease [9], and inflammation, due to collagen disease [10], are involved in the development of PAH [11]. These factors interact with each other in a complex manner and affect pulmonary vasculature continuously [1,12]. Thereby, pulmonary artery smooth muscle cells (PASMCs) will suffer epigenetic modifications by transcriptional factors, such as hypoxia-inducible factor-1α (HIF-1α) and forkhead box protein O3a (FOXO3a) [13]. Thus, the identification of pathogenic genes, which induce the abnormal characteristics of PASMCs, should be useful for the development of novel therapies for PAH.

The characteristics of PASMCs of patients with PAH (PAH-PASMCs) are different from those of healthy controls [14,15]. Recently, it has been demonstrated that the abnormal features of PAH-PASMCs are based on their mitochondrial dysfunction [16,17,18,19]. These features of PAH-PASMCs may be caused by some unknown pathogenic genes that promote PAH [20]. Here, we hypothesized that the pathogenic proteins in PAH-PASMCs could be secreted and detected in circulating blood, affect remote organs, and cause dysregulation of systemic metabolism.

In this review, we would like to introduce the recent progress on the basic and clinical research focusing on the screening of pathogenic proteins in PAH [21].

## 2. Crucial Roles of AMP-Activated Protein Kinase (AMPK) Against PAH

Cytokines/chemokines and growth factors regulate pulmonary endothelial function and influence the development of PAH [22]. Endothelial dysfunction is a crucial pathogenic status that triggers a variety of vascular disorders, such as PAH [23,24]. Endothelial dysfunction is also considered a key underlying feature in most forms of clinical and experimental PAH, which is enhanced by inflammatory cytokines/chemokines and growth factors [21,25,26]. We have revealed a protective role of the endogenous erythropoietin (Epo)/Epo receptor (EpoR) system against the development of pulmonary hypertension (PH) [27]. This system also plays a crucial role in the functional recovery of ischemic heart [28] and ischemic lower limb [29], demonstrating the importance of endothelial function and homeostasis [30,31]. Moreover, we found that pravastatin and metformin protect pulmonary endothelial function and ameliorate hypoxia-induced PH in animals [25,32].

AMP-activated protein kinase (AMPK) is an evolutionary conserved serine/threonine kinase that functions as an important energy sensor [33] and is activated by inhibition of Rho-kinase [34], which plays a crucial role for PAH [35,36,37]. AMPK has an anti-apoptotic effect in endothelial cells [38] and a pro-apoptotic effect in vascular smooth muscle cells (VSMCs) [39], which are critical for vascular remodeling. Both endothelial nitric oxide (NO) production and NO-mediated signaling in VSMCs are targets and effectors of the AMPK signaling pathway [33]. In endothelial cells, AMPK positively regulates NO production. In VSMCs, AMPK reduces intracellular signaling and secretion of many growth factors, promoting VSMC proliferation and vascular remodeling [33]. We have recently demonstrated that endothelial AMPK plays an important role in microvascular homeostasis and regulation of systemic arterial pressure in mice in vivo [40]. AMPK activators, such as statins, metformin, and apelin are protective against PAH [32,41,42,43,44]. Consistently, we have demonstrated that endothelial AMPK plays protective roles against hypoxia-induced PH in mice [25,32]. There are several medications and compounds to activate endothelial AMPK signaling in vivo, including salicylate and methotrexate [45,46]. Salicylate is an ancient drug, which is the major breakdown product of aspirin [47]. The low-dose of aspirin exerts anti-platelet effects in patients with coronary artery disease (CAD), which contributes to the significant improvement of long-term survival in CAD patients [48]. When we consider these backgrounds [45,47], it could be possible that the efficacy of aspirin in CAD patients is partially due to its stimulatory effect on endothelial AMPK signaling. Thus, AMPK modulators may exert effectiveness in patients with PAH. Our results suggest the potential role of circulating inflammatory cytokines for inducing endothelial dysfunction in pulmonary circulation [25]. Thus, endothelial AMPK, as well as circulating inflammatory cytokines, may be therapeutic targets for the treatment of PAH. Indeed, increased serum levels of cytokines in inflammatory status contribute to the acute progression and worsening of clinical status in PAH patients [49]. Thus, AMPK is a key molecule at the crossroad of inflammation and pulmonary artery endothelial dysfunction in the pathogenesis of PAH.

## 3. Crucial Roles of CyPA and Bsg in the Development of PAH

Hypoxia induces activation of NFAT (nuclear factor of activated T cells) and promotes VSMC proliferation [50]. Chronic hypoxic exposure of mice induces vascular remodeling characterized by medial and adventitial thickening of the muscular and elastic vessels and muscularization of more distal small vessels [51]. Pulmonary vascular inflammation plays a crucial role in the development of hypoxia-induced PH [27,32], for which Rho-kinase plays a crucial role [35,36,37]. Additionally, Rho-kinase promotes secretion of cyclophilin A (CyPA) from VSMCs and extracellular CyPA stimulates VSMC proliferation in vivo [52] and in vitro [53,54]. CyPA is secreted from VSMCs through Rho-kinase activation [55]. Extracellular CyPA induces endothelial cell adhesion molecule expression [56], induces apoptosis [57] and is a chemoattractant for inflammatory cells [52,58]. Basigin (Bsg, also known as CD147 or EMMPRIN) is an extracellular CyPA receptor [59]. Importantly, Bsg is an essential receptor for Malaria, which disrupts NO metabolism and causes harmful endothelial activation, including the Rho/Rho-kinase activation [60]. Consistently, we have demonstrated that CyPA and Bsg contribute to hypoxia-induced PH [61]. *CyPA*^+/-^ and *Bsg*^+/-^ mice exhibited resistance to hypoxia-induced pulmonary vascular remodeling. Moreover, plasma CyPA was significantly increased in patients with PAH and well correlated with disease severity and long-term survival. Thus, extracellular CyPA and its signaling through Bsg are novel therapeutic targets for PAH. We further propose a key role for CyPA/Bsg signaling in pulmonary vascular remodeling. Specifically, we propose that hypoxia-induced secretion of growth factors and cytokines/chemokines requires CyPA/Bsg signaling in the pulmonary vasculature. Indeed, recent in vivo studies showed that Bsg in circulating inflammatory cells functions as a CyPA receptor [62,63]. Consistently, Bsg expression was intense in the perivascular inflammatory cells of animal models of PH and patients with PAH [61]. Bsg induces Rac1-dependent expression of inflammatory cytokines [64] and promotes VSMC proliferation [65]. These reports support our notion that the secretion of inflammatory cytokines was augmented by cooperative interaction between extracellular CyPA and Bsg in the pulmonary vasculature. A key aspect of this study is the strong expression of CyPA and Bsg in the pulmonary arteries of animal models of PH and patients with PAH. We have previously reported that statins and Rho-kinase inhibitors reduce CyPA secretion from VSMCs [66,67], Rho-kinase is an important therapeutic target in cardiovascular diseases [68] and Rho-kinase inhibition ameliorates PH in animals and humans [69,70,71,72]. Thus, inhibition of CyPA secretion by Rho-kinase inhibitors may have contributed to the therapeutic efficacy of these drugs in PAH [69,70]. In addition, Bsg stimulates MMP production [73]. Importantly, Bsg is strongly expressed in the pulmonary arteries of patients with PAH [61]. Thus, it is logical to consider that that pharmacological agents that prevent the interaction of extracellular CyPA and vascular Bsg could be useful for the treatment of PAH. The identification of CyPA as a novel biomarker and mediator of PH associated with inflammation provides insight into the mechanisms of several therapies. CyPA has been found as a binding partner of cyclosporine A (CsA), which is an immunosuppressive drug in clinical use [74]. It has been established that the CyPA-CsA complex binds to and inactivates calcineurin, which activates nuclear factor of activated T cells (NFAT) transcription factors [75]. Since this step is important for cytokine/chemokines production and secretion, inhibition of calcineurin by CsA exerts anti-inflammatory effects. Here, there is strong evidence of an important role of NFAT in PAH-PASMCs and the infiltrating inflammatory cells [76,77,78]. Thus, there is a potential Bsg-independent role of intracellular CyPA on NFAT activation in the development of PAH.

## 4. Screening of Inhibitors for CyPA and Bsg

Heart failure (HF) has been emerging as a pandemic health issue worldwide [79]. Furthermore, severe HF is accompanied by post-capillary PH, which is characterized by impaired pulmonary vascular reactivity, endothelial dysfunction, and distal pulmonary artery muscularization [80,81]. Once post-capillary PH develops, HF patients show more severe symptoms, worse exercise tolerance, and poor prognosis [80,82]. Some possible treatments for post-capillary PH have been tested in animal models of HF [83,84]. While targeting both cardiac dysfunction and post-capillary PH could be a promising therapy for HF patients, therapeutic targets that share molecular mechanisms of both diseases need to be explored. Recently, our drug discovery research demonstrated that celastrol significantly inhibits CyPA and Bsg, improving pressure-overload-induced cardiac hypertrophy and post-capillary PH in mice [85]. Importantly, celastrol suppressed CyPA and Bsg expressions in the heart and lung and ameliorated both HF and post-capillary PH. Interestingly, CyPA and Bsg play critical roles as downstream targets of Rho-kinase in the enhancement of ROS production. CyPA is one of the causative proteins that mediate oxidative stress-induced cardiovascular dysfunctions, such as atherosclerosis, abdominal aortic aneurysm, and cardiac hypertrophy [57,67,86]. Furthermore, one of the CyPA receptors, Bsg, also plays crucial roles in the pathogenesis of PH, cardiac hypertrophy, and HF [61,87]. Our recent study demonstrated a synergy between Rho-kinase and CyPA to increase ROS generation [88]. As ROS stimulates myocardial hypertrophy, matrix remodeling, and cellular dysfunction [89], Rho-kinase (especially ROCK2) and CyPA promote ROS production, as well as cardiac hypertrophy and failure in a synergistic manner. Consistent with our recent studies, we detected a synergy between Rho-kinase, CyPA, and Bsg to increase ROS production. Thus, Rho-kinase, especially ROCK2, and CyPA may promote ROS production, as well as cardiac hypertrophy and failure in a synergistic manner. Both CyPA and Bsg are known to accelerate PH by stimulating oxidative stress and inflammation [61,90]. Similarly, CyPA and Bsg may exacerbate post-capillary PH by stimulating oxidative stress and inflammation. Taken together, inhibiting both CyPA and Bsg may represent a novel therapeutic strategy for the treatment of HF patients with post-capillary PH [85]. As patients with HF and coexisting post-capillary PH show poor clinical outcomes [80,82], targeting both cardiac dysfunction and pulmonary vascular remodeling could be a novel concept for the treatment of HF. We and others demonstrated that fasudil, a specific Rho-kinase inhibitor, is effective in animal models of HF [91,92,93,94,95]. In contrast, we previously demonstrated that Rho-kinase inhibition in mice by SM22α promoter-driven overexpression of dominant-negative Rho-kinase showed arrhythmogenic right ventricular cardiomyopathy [96]. This result indicates that long-term isoform non-selective inhibition of Rho-kinase may have an impact on cardiac function. Indeed, we showed that pressure-overload-induced cardiac dysfunction and post-capillary PH were accelerated in *ROCK1*^–/–^ mice compared with littermate controls, suggesting that ROCK1 plays a crucial role to maintain cardiac function in loaded conditions [85]. In contrast, *ROCK2*^–^^/–^ mice showed decreased cardiac hypertrophy compared with littermate controls after pressure-overload. Furthermore, ROCK2 in cardiac fibroblasts is necessary to cause cardiac hypertrophy and fibrosis [97]. It has recently been demonstrated that a selective ROCK2 inhibitor, KD025, could be an effective treatment for ischemic stroke and autoimmune diseases [98,99,100]. The present study indicates that selective ROCK2 inhibition could be a more favorable therapy for HF. However, cardiomyocyte-specific ROCK2-deficient mice showed a slight, but not dramatic improvement in cardiac hypertrophy and fibrosis under pressure-overload [85]. Moreover, when we consider the complex interactions between ROCK1 and ROCK2 in cardiomyocytes and other cell types, the use of selective ROCK2 inhibitor may not meet the clinical needs to cure patients with HF. Thus, we focused on CyPA and Bsg as common molecules that augment HF and PH. We used PASMCs and high-throughput screening to identify novel agents to inhibit both CyPA and Bsg. In the present study, we aimed to develop a novel therapeutic agent by focusing on both CyPA and Bsg, two downstream targets of Rho-kinase [101]. As an additional strategy for HF with post-capillary PH, effective treatment that achieves inhibition or reverses remodeling of pulmonary arteries is warranted [102]. PASMCs in the remodeled pulmonary arteries have special characteristics with pro-proliferative features. Based on the development of academic drug discovery, we focused on the inhibition of PASMC proliferation to discover a novel drug for HF with post-capillary PH. We performed phenotypic screening and discovered compounds with inhibitory effects on CyPA and Bsg. We finally selected celastrol, presenting with anti-oxidant effects, for in vivo treatment. Celastrol improved pressure-overload-induced cardiac dysfunction and post-capillary PH with no apparent side effects, suggesting that inhibiting the proliferation of PASMCs may be a novel therapeutic strategy to treat HF patients with post-capillary PH. Thus, celastrol may be a promising drug for HF. Celastrol is a compound obtained from *Tripterygium wilfordii* and its usefulness has been reported in some inflammatory diseases, such as rheumatoid arthritis, systemic lupus erythematosus, inflammatory bowel diseases, osteoarthritis, and allergy [103]. Celastrol suppresses the activity of nuclear factor kappa B (NF-κB), which upregulates inflammatory genes and enhances cardiac hypertrophy [104] and pulmonary vascular remodeling [105]. As CyPA and Bsg activate NF-κB [90,106], the effect of celastrol on HF may have been due to the inhibition of CyPA/Bsg-NF-κB axis, which enhances ROS generation and inflammatory status.

## 5. Identification of Novel Therapeutic Targets for PAH

**Figure 1:**
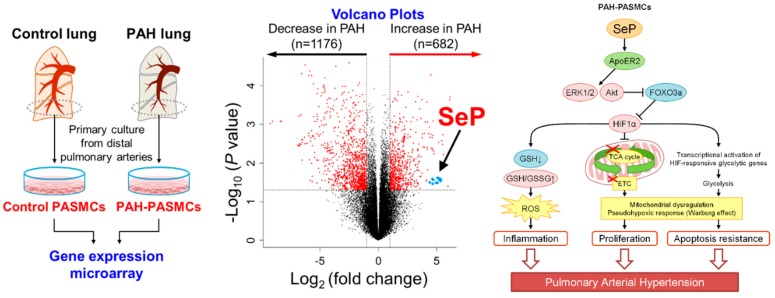
Screening of novel therapeutic targets for pulmonary arterial hypertension and schematic representation of the role of selenoprotein P (SeP). Volcano plots of gene expression variations in PAH-PASMCs and control PASMCs. Blue plots represent probes for SeP. Dashed lines represent an adjusted P value of 0.05 and ± 1-fold change. PAH-PASMCs, pulmonary artery smooth muscle cells harvested from patients with pulmonary arterial hypertension (PAH); ApoER2, apolipoprotein E receptor 2; ERK1/2, extracellular signal regulated kinases 1 and 2; ETC, electron transport chain; FOXO3a, forkhead box protein O3a; GSH, glutathione; GSSG, oxidized glutathione; HIF-1α, hypoxia inducible factor 1α; PAH-PASMCs, pulmonary arterial hypertension-pulmonary artery smooth muscle cells; ROS, reactive oxygen species; SeP, selenoprotein P; TCA cycle, tricarboxylic acid cycle.

To identify a novel pathogenic protein, we first performed microarray analyses using PAH-PASMCs and found 32-fold upregulation of selenoprotein P (SeP), as compared with control PASMCs [107] (Figure 1). SeP is a secreted protein mainly produced by hepatocytes, but also detected in many types of cells [108,109]. SeP contains 10 selenocysteine residues and transports selenium to maintain cellular redox state and metabolism [110,111,112,113]. Recently, it has been reported that SeP is upregulated in the liver of patients with type 2 diabetes, and downregulates the metabolic switch, AMPK [114]. Moreover, single nucleotide polymorphisms in the *SEPP1* gene have been reported to be associated with abdominal aortic aneurysm formation [115]. These findings suggest that SeP regulates cellular metabolism and the development of vascular diseases. Here, we have shown that SeP in PASMCs promotes cell proliferation through increased oxidative stress and mitochondrial dysfunction in an autocrine/paracrine manner [107]. In addition, using five strains of genetically modified mice, we demonstrated a pathogenic role of SeP in the development of hypoxia-induced PH. Finally, we identified that sanguinarine, an orally active small molecule, reduces SeP expression and PASMC proliferation, and ameliorates PH in mice and rats. Although it has been shown that 60% of serum SeP is produced by hepatocytes in the physiological condition [108], SeP is also expressed in many types of cells for secretion [115,116,117]. Indeed, we demonstrated that SeP is highly upregulated in the distal pulmonary arteries of PAH patients. Our findings suggest that the upregulation of SeP in PAH-PASMCs is a trigger, as well as a promoter, for the development of PAH. We also demonstrated that SeP-mediated PASMC proliferation may mechanistically involve HIF-1α-mediated mitochondrial dysfunction, similar to cancer cells [118]. Indeed, activated HIF-1α in normoxia is well known in PAH-PASMCs, which induces the transcription of many genes producing pro-proliferative and anti-apoptotic signals, impaired oxidative glucose metabolism, and the shift to aerobic glycolysis [119]. In PAH-PASMCs, we found that the expression of SeP and HIF-1α affected each other, which accompanied SeP-mediated activation of Akt, ERK1/2, and resultant FOXO3a phosphorylation and degradation. Additionally, we found that serum levels of selenium were increased in PAH patients, suggesting that SeP function as a selenium supplier is preserved in PAH patients. Moreover, overexpression of mutated SeP, which has no selenium, also increased ROS levels and reduced GSH in control PASMCs, which was similar to the effect by overexpression of intact SeP. Indeed, it has been reported that SeP possesses two functions; the enzyme activity in the N-terminal region and the selenium-supply activity in the C-terminal region [120]. Additionally, ApoER2 is a candidate receptor for SeP in PAH-PASMCs and activates intracellular signaling pathways [121]. Altogether, our findings on selenium status in PAH patients and previous reports indicate that SeP-mediated development of PAH is independent of its selenium supply. Here, we found that the enhanced expression of SeP reduced the GSH/GSSH ratio and increased oxidative stress levels, contributing to the enhanced expression and stabilization of HIF-1α even in normoxia and these changes had no relation to selenium content in SeP. In addition to these findings, we found that *Sepp1^–/–^* PASMCs had increased GSH/GSSG ratio, reduced ROS levels, and HIF-1α levels in both normoxia and hypoxia. These results suggest that SeP, even without its selenium content, acts as an upstream negative regulator of antioxidative stress signaling, which induces both ROS generation through NADPH oxidases and stabilizes HIF-1α, providing a potential mechanism of SeP-mediated HIF-1α activation and resultant proliferation of PASMCs and their survival in PAH.

SeP promoted PASMC proliferation, which prompted us to find a SeP inhibitor as a novel therapy for PAH. At this point, we have no drugs available for targeting PASMC proliferation [122], and a limited report of SeP targeting, in which metformin may suppress its expression in the liver via AMPK activation [123]. Coincidentally, we have recently demonstrated that endothelial AMPK plays a crucial role in suppressing the development of hypoxia-induced PH, which effect can be achieved with metformin [25]. However, metformin has no effect on the expression of SeP in PAH-PASMCs (unpublished observation). Moreover, we performed in silico screening and found no compound with an inhibitory effect on SeP. It has been reported that oral sanguinarine administration successfully inhibited tumor growth [124]. When we consider the pro-proliferative role of SeP in PAH-PASMCs, the anti-proliferative effect of sanguinarine in several kinds of cancer in vivo could be attributed to the suppression of SeP [124]. Actually, sanguinarine administration to the animal models of PH revealed therapeutic effects on PH and RV failure without any adverse effects [107]. Moreover, serum levels of SeP were significantly elevated in PAH patients, in whom higher serum levels of SeP predicted a poor outcome. Conversely, treatment with SeP inhibitors reduced protein levels of SeP and ameliorated PH. Based on these results, serum levels of SeP can be used as a novel biomarker for PAH and are useful to evaluate the therapeutic effect of SeP inhibitors (companion diagnostics). Using a combination of SeP inhibitors and serum levels of SeP, we may find good candidates among PAH patients that can be used to demonstrate the effectiveness of this strategy. By targeting SeP, we will promote translational research and develop early diagnostics and novel therapeutic agents for the treatment of PAH patients.

## 6. TAFI as a Novel Therapeutic Target for CTEPH

In addition to PAH, we have recently demonstrated that thrombin-activatable fibrinolysis inhibitor (TAFI) is a novel biomarker for patients with chronic thromboembolic pulmonary hypertension (CTEPH) [125,126]. TAFI is a glycoprotein that is cleaved and activated by the interaction with thrombin and thrombomodulin (TM) in vascular beds [127,128]. The emergence of balloon pulmonary angioplasty (BPA) significantly improved the prognosis of patients with CTEPH [129,130,131,132]. The main feature of CTEPH is obstruction of pulmonary arteries by organized thrombi [129,133]. Since the pathogenesis of CTEPH has been unclear for a long time, we have attempted to find a key molecule to elucidate the pathogenesis of this disorder. We found that plasma levels of TAFI were significantly elevated in CTEPH patients and were unaltered even after hemodynamic improvement [125,126]. Additionally, we found the minor allele *CPB2* in CTEPH patients compared with the general population [125]. Moreover, plasma levels of activated TAFI (aTAFI) were negatively correlated with clot lysis time in CTEPH patients [125]. Thus, to evaluate the effects of aTAFI inhibition, we performed in silico screening using the Life Science Knowledge Bank (LSKB) database and found several aTAFI inhibitors, and one of them ameliorated the development of PH in mice [126]. Additionally, we found that peroxisome proliferator-activated receptor-α (PPARα) agonists significantly reduced liver TAFI synthesis and ameliorated PH in mice and rats [126]. Based on the basic research, we have started to plan clinical research by using a PPARα agonist in patients with CTEPH. Thus, aTAFI could be a novel and realistic therapeutic target of CTEPH.

## 7. Conclusions

During the past few decades, increased understanding of PAH pathophysiology has led to the development of several effective therapies, including prostacyclin (PGI2) analogues and derivatives, endothelin receptor antagonists, phosphodiesterase type 5 (PDE5) inhibitors, and a soluble guanylate cyclase (sGC) stimulator. In this Review article, we introduced our recent findings on Epo, AMPK, Rho-kinase, CyPA, Bsg, and SeP, all of which are substantially involved in the pathogenesis of PAH (Table 1). Additionally, we have also mentioned as to the screening of inhibitors for those pathogenic proteins. By using the novel biomarkers and therapeutic agents, we will continue translational research for the early diagnosis and the development of fundamental therapy in PAH patients.

## Figures and Tables

**Table 1:** New candidate molecules for novel targets in pulmonary hypertension.

New Candidate Molecules	Novel Targets	References
Fasudil	Rho-kinase (human, inharation)	[134]
Fasudil	Rho-kinase (human, oral)	[72]
Fasudil	Rho-kinase (rodents)	[37]
Metformin	AMPK (mouse)	[25]
PPARα agonist (Fenofibrate, WY14643)	TAFI (mouse)	[126]
TAFIa inhibitor (carboxypeptidase inhibitor)	TAFI (mouse)	[126]
Sanguinarin	Selenoprotein P (rat)	[107]
Celastrol	Cyclophilin A and Basigin (mouse)	[85]
